# Role of Microglia in Age-Related Changes to the Nervous System

**DOI:** 10.1100/tsw.2009.111

**Published:** 2009-10-02

**Authors:** David R. Brown

**Affiliations:** Department of Biology and Biochemistry, University of Bath, United Kingdom

**Keywords:** prion, amyloid, neurodegeneration, iron, microglia, neuron

## Abstract

Microglia play a curious role in the nervous system. Their role is intrinsically protective and supportive, but during neurodegenerative disease, it is well established that microglia play a significant role in the initiation of neuronal death. Microglia, like neurons, show age-related changes that could potentially alter their behavior. While extreme changes to a large population of microglia cause dramatic neuronal loss in neurodegeneration, during normal aging, subtle changes not unlike those seen in the disease state could potentially contribute to a more gradual neuronal loss that could contribute to the cognitive decline seen in the aging population. This review provides illustrations of what is known about the role of microglia in neurodegeneration and makes suggestions about the role of microglia in age-related changes to the brain.

## INTRODUCTION

It is now well accepted that glia are an integral part of the central nervous system and play a critical role in homeostasis in the brain. Neuronal aging or degeneration still remains largely the central focus of much research. Some amount of interest in changes to glia has also developed. Possibly, further consideration needs to be given to the concept that glia will also age and show changes that then impact upon neuronal viability. This concept is somewhat different to that proposed in the study of neurodegeneration where a series of changes to key proteins result in altered neuronal ability to resist various toxic insults and, in the process, glia also become compromised and their altered phenotype then contributes to the accelerated neuronal loss observed. The difficulty, as always with the understanding of normal aging, is in generating a model that can be studied in the short-term scales employed in research.

As microglia have a greater capacity to take on a pathological phenotype, possibly as a result of their extraneural origin, they are then a more likely culprit for age-dependent changes that could have knock-on effects for neurons. This is clearly evident from the study of neurodegenerative disorders where their involvement in neuronal loss has been demonstrated. In the adult brain, resting microglia exhibit quiescent, ramified morphology. Adult microglia become activated in response to brain injuries or immune challenge[1,2,3,4]. Upon activation, microglia undergo morphologic alterations, changing from resting, ramified microglia into activated microglia[1]. Further, surface molecules, such as complement receptors and major histocompatibility complex molecules, are also up-regulated when microglia are activated and they can transform into phagocytic macrophages[5,6]. In addition, activated microglia are capable of releasing a variety of soluble factors, which are proinflammatory in nature and potentially cytotoxic. Such cytotoxic factors include superoxide[7], nitric oxide (NO)[8,9], and tumor necrosis factor alpha (TNFα)[10,11]. These changes in microglia have been observed in a number of neurodegenerative conditions, such as Alzheimer's and prion diseases[12,13]. There is also evidence of increased microglial production of proinflammatory cytokines during aging[14]. In contrast to this deleterious role, microglia are also reported to increase neuronal survival through the release of trophic and anti-inflammatory factors[15,16,17]. Thus, rather than classify microglia as exclusively beneficial or inherently deleterious, it is likely that microglia can play alternative roles, depending on differing conditions within the brain. Thus, in neurodegenerative disease and possibly aging, microglia play a role as mediator rather than an initiator of neuronal changes. In this regard, understanding the role of microglia and preventing their activation could lead to strategies to alleviate neuronal damage and loss without necessarily defining or eliminating the root cause.

## NEURODEGENERATION AND MICROGLIA

Reliable models of aging are difficult to develop and the relevance of existing models remains unclear. However, in the study of neuronal loss and damage during aging, the mechanisms involved are likely to have some relation to the mechanisms by which neurons are similarly affected in neurodegenerative diseases. Neurodegenerative diseases are age-related diseases for the most part, and possibly involve an acceleration of ongoing processes. Therefore, understanding the potential role of microglia in these diseases is of considerable benefit for the study of age-associated neuronal changes.

The number of diseases where a role for microglia has been suggested continues to increase[18,19,20]. The first and most obvious way that microglia have been implicated in neurodegeneration was the observation of gliosis in the brains of patients with a variety of diseases. The observation of proliferation and activation of microglia clearly implies that they either react to or could potentially cause a change in the brain. A change in the brain tightly associated with microglial activation is the process of demyelination. In particular, demyelination is associated with multiple sclerosis (MS). There is a clear inflammatory component involved in MS, even though the cause of the disease is unknown. Lymphocytes and activated myeloid cells are localized in the area of demyelization[21,22,23]. Analysis of the brains of MS patients using nuclear magnetic resonance imaging, positron emission tomography, and [11C](R)-PK11195 (a microglial marker) showed increased microglial activity around the site of the MS lesion[24]. The role of microglia in MS is also supported in the MS animal model of experimental autoimmune encephalomyelitis, where microglia are shown to proliferate and increase lysosome activity around active sites of demyelization[25]. Once at the site of lesion in MS, microglia increase expression of cyclooxygenase 2[22] and inducible nitric oxide synthase (iNOS)[21]. Both of these proteins are associated with cytotoxic effects of microglia. In addition to being a source of neurotoxic factors upon activation, microglia have been implicated in the initiation and progression of MS as one of the antigen-presenting cells that sparks the autoimmune response targeting myelin[26]. Thus, while infiltrating T cells and macrophages have a clear role in MS-associated demyelination, lesions, and neuronal damage, microglia are also critically involved in this process.

Microglia have also been associated with a range of diseases due to their ability to secrete proinflammatory substances, such as cytokines. These diseases include hypoxia[27], stroke[28], amyotrophic lateral sclerosis[24,29], neuropathic pain[30], and Parkinson's disease[31]. However, despite the similar responses of microglia, the results of the diseases have diverse pathologies. In Parkinson's disease, there is a specific loss of dopaminergic neurons from a region of the brain termed the substantia nigra. Dopaminergic neurons are inherently susceptible to the damaging effects of microglial activation. This selective mechanism of microglia-mediated dopaminergic neurotoxicity is possibly due to the generation of oxidative substances by activated microglia. In particular, dopaminergic neurons possess reduced antioxidant capacity, as evidenced by low intracellular glutathione, which renders dopaminergic neurons more vulnerable to oxidative stress and microglial activation relative to other cell types[32].

While oxidative stress is clearly toxic to multiple cell types, dopaminergic neurons may succumb first at lower levels of oxidative stress, followed by other neuronal and cell populations. Additionally, the substantia nigra contains 4.5 times as many microglia when compared to the cortex and other regions of the brain[33], suggesting that the localization of microglia in the substantia nigra predisposes them to vulnerability to immunological insult.

Microglia-mediated cell death via cytokine release or the generation of oxidative stress is the most common mechanism by which microglia induce neuronal death. The study of neurodegeneration with models has proven difficult because, as with aging, animal models are difficult to generate or do not exist. One of the ways researchers have attempted to overcome this is to identify compounds that induce similar changes in animals as occur in human diseases. Substances that have been utilized this way include rotenone, lipopolysaccaride (LPS), and paraquat[34,35,36]. All three substances caused specific loss of dopaminergic neurons and for this reason have been utilized in models for Parkinson's disease. The action of these substances is mediated through microglia, highlighting the importance of these cells to neurodegeneration[35,37,38]. While the use of these substances is artificial and does not cause the diseases they model, the implication of these findings is clearly that changes to microglia have significant consequences for neuronal survival. Therefore, in aging, where microglial changes might be similar, even if more subtle, then the same mechanisms are likely to be active in terms of damaging or killing neurons.

While the focus of this review is microglia and aging, the relevance of changes in neurodegeneration to consideration of the changes that occur in aging makes it important to consider some details of the involvement of microglia in specific cases of neurodegenerative disease.

## MICROGLIA IN PRION DISEASE

Prion diseases or transmissible spongiform encephalopathies are the single example of a neurodegenerative disease where the disease can be transmitted between individuals. It is also the only neurodegenerative disease where natural animal models exist. Prion diseases have three main varieties[39]. Inherited diseases, such as Gerstmann-Straüssler-Scheinker syndrome (GSS), exist where point mutations in the prion protein gene are associated with onset of disease in humans at approximately the age of 50. There are also sporadic forms of the disease, such as the sheep disease scrapie and the main human form, Creutzfeldt-Jakob disease (CJD). In these cases, disease occurs spontaneously with no known cause. Lastly, there are the laterally transmitted forms. Variant CJD is largely considered to be one of these as it is likely to have been transmitted to humans through the consumption of BSE-contaminated food.

The existence of animal models has made the study of prion disease relatively straightforward, as the time course of the latent phase of the disease, otherwise termed the incubation period, can be studied due to the high reproducibility of the time course of symptoms and pathological changes that occur with the use of defined strains of scrapie. There has been considerable study of the role of microglia in prion diseases[40,41]. These studies have involved the analysis of tissue from the brains of patients[42], studies on scrapie-infected mice[43], and studies using cell-based systems[13]. Studies with mice have been particularly important in demonstrating that microglial activation and proliferation occur prior to significant neuronal loss, but subsequent to accumulation of the abnormal PrP isoform ($PrP^{Sc}$) in the brains of the mice[44,45].

Experimental evidence for the role of microglia in neuronal loss in prion disease models came from *in vitro* studies using the coculture of microglia and neurons[13,45]. In these studies, toxicity of $PrP^{Sc}$ or a peptide mimic, PrP106-126, only showed toxicity when microglia were present. The toxicity was shown to be a result of superoxide generated by microglia and a loss of cellular resistance to oxidative stress. Microglia also respond to $PrP^{Sc}$ and PrP106-126 with phenotypic changes, such as rapid proliferation, activation, and the release of cytokines, which are both proinflammatory and able to induce astogliosis[46,47,48,49,50]. Studies in mice have shown that prion infection alters neuronal expression such that they are able to activate microglia, which could induce neuronal loss when activated[51].

Changes to neurons also include effects such as the release of substances that activate microglia[52]. Thus, either directly or indirectly, microglia recruited to the vicinity of neurons due to the presence of prions can cause neuronal death.

The study of prion disease models has highlighted the potential of cell-cell interactions in the deleterious changes to neuronal health (Fig. 1). Such interactions between microglia and neurons, or even more complex loops, such as microglia, astrocytes, and neurons, result in very specific local changes that initiate neuronal damage. In the case of models of prion disease, these involve the exchange of molecules between microglia and neurons, such a cytokines. With the additional involvement of astrocytes, microglia release substances that cause astrocyte proliferation and/or the release of a substance, such as interleukin-6, which has additional effects on neurons other than those directly between neurons and microglia[48]. Additionally, the presence of a large number of astrocytes, as a result of stimulated proliferation by microglia, can alter the toxicity of other substances, such as glutamate, which are then able to induce further neuronal loss[53]. There is some evidence that microglia in aging mice show an altered behavior in which they are more likely to produce proinflammatory cytokines and other potential toxic molecules[54]. This altered cytokine production does not appear to be associated with an abnormal stimulation[55]. The implication is that aging changes mimic changes seen in diseases like prion disease and could contribute to age-related changes to the central nervous system. There is also evidence that secretion of some proinflammatory cytokines by microglia could injure neurons without necessarily leading to immediate neuronal loss, which could also contribute to cognitive decline in the aging brain[56].

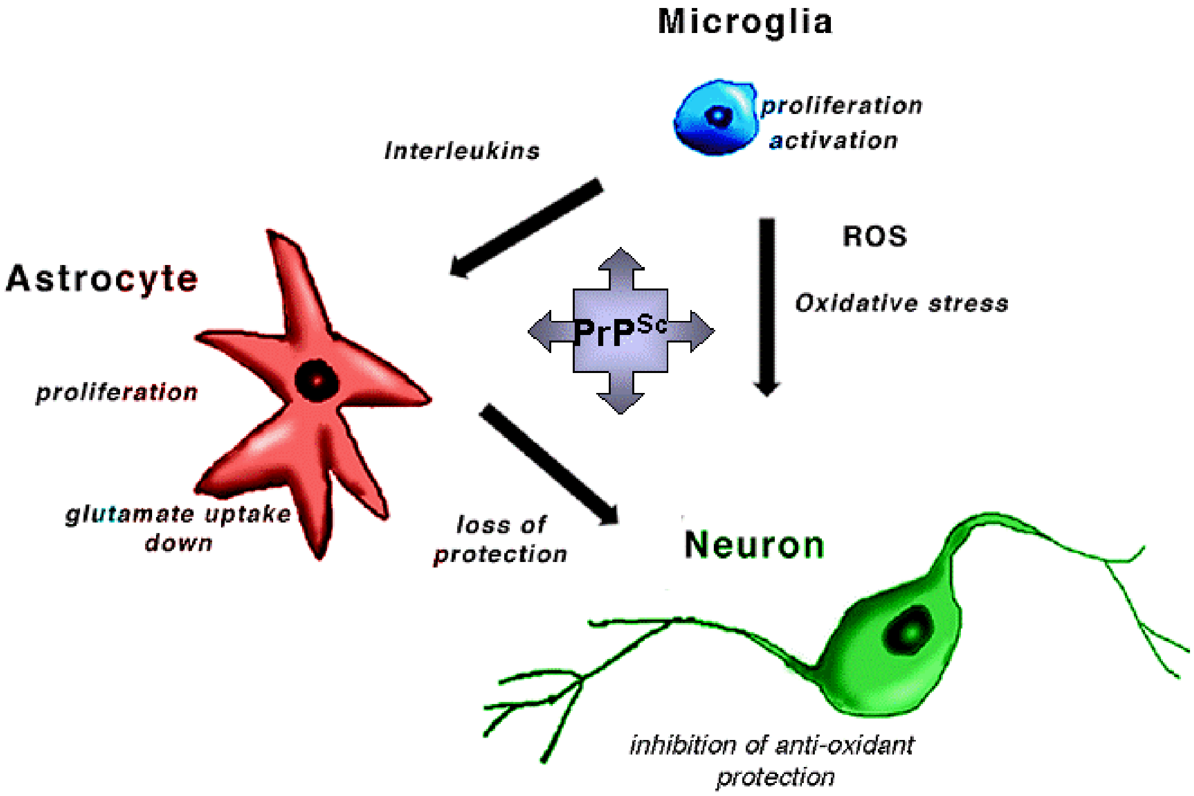

**FIGURE 1.** Schematic representation of the interaction of neurons and microglia in a model of prion disease. The abnormal isoform of the prion protein ($PrP^{Sc}$) is generated during the course of prion disease. It is considered both the infectious agent and the cause of neuronal loss. Its neurotoxicity results from a mixture of direct and indirect effects. $PrP^{Sc}$ causes a reduction in neuronal resistance to oxidative stress, but also causes activation of microglia and stimulates their proliferation. The production of reactive oxygen species (ROS) and cytokines from the microglia has significant knock-on effects, causing stimulation of astrogliosis and toxicity to neurons. $PrP^{Sc}$ also inhibits astrocyte clearance of glutamate, further increasing neuronal loss.

## MICROGLIA IN ALZHEIMER'S DISEASE

Alzheimer's disease (AD) is the most common neurodegenerative disease. The incidence increases with age such that those over 80 have a 45% chance of developing the disease. AD was one of the first neurodegenerative diseases associated with neurotoxic microglial activation. Amyloid-beta (Aβ) is tightly linked to AD and is a breakdown product of the amyloid precursor protein. Generation of Aβ is increased in some forms of inherited AD. A fraction of Aβ generated is slightly longer than the majority (Aβ1–42). This form, in particular, is associated with AD. Aβ aggregates to form deposits in the brain, including amyloid plaques. Deposition of Aβ in plaques is associated with both recruitment and activation of microglia[12,57,58]. Microglia associated with plaques have been reported to release neurotoxic substances, such as NO[5,59], TNFα[60], and superoxide[61]. Inflammatory responses in AD have been well documented and many of these potentially involve activated microglia[62].

Use of synthetic Aβ has determined that the amino acid residues 10–16 of the peptide are critical for microglial activation[63]. Several receptors have been implicated as necessary for the interaction of microglia and Aβ, such as the CD14 receptor[64] and the β1-integrin receptor[65]. Microglia interact with Aβ through cell surface receptor complexes consisting of the B-class scavenger receptor CD36, α6-β1 integrin, and CD47 (integrin-associated protein). Additionally, the receptor complex reported to be responsible for the internalization of Aβ (CD36, α6-β1 integrin, and CD47) has also been identified as critical for Aβ-induced superoxide production in microglia[66].

In animal models of AD, inhibition of glial activation attenuates neurotoxicity. Infusion of human Aβ1–42 into rodents replicates many of the hallmarks of AD pathology (neuroinflammation, neuronal and synaptic degeneration, and amyloid deposition). Administration of aminopyridazines was shown to both attenuate glial inflammation and result in reduction of neuronal neurotoxicity in the rodent intraventricular human Aβ1–42 infusion model[67]. This supports the concept that therapeutic inhibition of Aβ-induced inflammation could be neuroprotective. In a separate study, nonsteroidal anti-inflammatory drug (NSAID) treatment in mice overexpressing Aβ was able to lower Aβ deposition, inhibit microglial activation, and provide neuroprotection[68], also indicating that inflammation induced by Aβ contributed to neurotoxicity. *In vitro* studies also support that Aβ is proinflammatory, where inhibition of synthetic Aβ-induced microglial activation with dextromethorphan[69] results in a reduction of Aβ-induced neurotoxicity. Clinical studies have investigated the effects of anti-inflammatory therapy for cognitive decline in AD patients, with some benefits being demonstrated[70,71]. A protective role of NSAIDs for AD therapy remains controversial[72]. Regardless of the outcome of certain drug trials, these studies indicate that inflammation and microglia are critical for the ongoing process of neurodegeneration in AD.

The role of microglia in AD clearly points to an interaction with plaques. A recent study has indicated that in the healthy brain, microglia play a role in the normal breakdown and clearance of the Aβ peptide[73]. In this case, the peptide taken up is proteolytically degraded. This clearly implicates microglia in the normal homeostasis of Aβ. It is therefore likely that changes in microglial behavior in AD relate to the altered nature of Aβ when in plaques. Despite the abundance of activated microglia in AD, they are inefficient in clearing Aβ deposits[74]. Older microglia show a decreased ability of phagocytosis Aβ[75]. This implies that a change in microglial behavior due to aging alone could result in increased accumulation of Aβ. The implication of this is that changes associated with normal aging could predispose older people to AD or be a contributing factor in the manifestation of the disease.

## DYSTROPHIC MICROGLIA AND AGING

Suggestion of a causal role for microglia in age-related changes in the central nervous system and cognitive decline requires evidence for age-related changes in microglia.

Currently, there is evidence for so-called "dystrophic" microglia present in the human brain with increased age. This is based on evidence of physical changes in microglia[76]. These changes include slight enlargement of the cell, distinct loss of fine branches (deramification), formation of cytoplasmic spheroids, gnarling, beading, and fragmentation of the cytoplasm. These changes were found to be widespread throughout the brain and not limited to specific regions. Evidence that these changes are important comes from the study of AD in which there is also an increased number of dystrophic microglia[77]. Dystrophic microglia could also be identified as they expressed high levels of ferretin, an iron storage protein[78]. The implication that damaged microglia are involved in iron storage suggests that their role in this capacity makes them susceptible to damage through the oxidizing action of iron. Iron storage by ferritin depends upon the oxidation state of the iron. Superoxide, which is produced and released by activated microglial cells[7,79], acts as a reducing agent and can therefore cause the release of iron from ferritin[80,81]. Superoxide-mediated iron leakage from ferritin has been shown to provoke membrane lipid peroxidation[80,81,82]. Studies of brain iron storage indicate that not only is there an increase of iron in the brain with age, but there is an increase in the proportion of iron stored in ferritin[83,84,85]. While there are many theories as to the cause of aging, the possibility that aging effects mediated through iron alter microglial behavior is an intriguing one. In this theory, it is not aging *per se* that causes changes to microglia, but the accumulation of increased iron. If these iron-accumulating microglia are the ones that then cause neuronal damage and then death, then there is the immediate possibility that aging in the brain and cognitive decline could be reduced by a form of chelation therapy that would selectively decrease the iron content of microglia. Such a possibility remains highly speculative at this time, but is an attractive prospect for further investigation.

## MICROGLIAL AGING

We have considered how microglia contribute to neuronal loss and hypothesized that microglia bring about this process in the aging brain due to a low-level production of toxic factors similar to that seen in neurodegenerative disease. Neurons might have an increased sensitivity to stress in the aging brain, but it is also possible that age-related changes in microglia play a more critical role.

As well as changes to morphology, iron storage, and cytokine production, there are a considerable number of other changes to microglia that occur as they age. The importance of any of these to neuron survival is unclear at present. These changes include increased accumulation of other substances, such as lipofuscin, and expression of proteins, such as cathepsins[86]. Another theory concerning microglia and aging relates to possible changes to microglial mitochondria. In this theory, mitochondria in microglia become increasingly damaged by oxidative stress within the cell. Mitochondrial DNA (mtDNA) is highly susceptible to the damage produced by reactive oxygen species (ROS) because of its close proximity to ROS generation through the respiratory chain and its paucity of protective histones. Furthermore, there is little capacity for DNA repair in mitochondria. During aging, a large number of mtDNA mutations accumulate in various tissues, including the brain, leading to dysfunction of the respiratory chain. As microglia generate large amounts of ROS and do so increasingly with age, then the potential for damage to mitochondria is increasingly greater for microglia than other cells. It is also known that as cells age, the autophagic process that removes damaged organelles, such as mitochondria, works less efficiently. The implication is that aging microglia would have more damaged mitochondria[87]. There is also a decline in the turnover of microglia with age, which implies that aging microglia are less likely to be replaced in the absence of gliosis-inducing factors[88]. It has been shown that overexpression of a mitochondrial protein, mitochondrial transcription factor A (TFAM), is able to reverse age-dependent deficits of learning and memory in mice[87]. This protein plays an important role in maintaining mtDNA by regulating the copy number of mtDNA and maintaining its higher structure[89]. This can protect mtDNA from ROS damage and improves respiratory chain function[90]. This highlights the potential role of mitochondria in healthy microglia and their potential role in microglial aging. Changes in the aging brain related to microglia are likely to be a result of a number of changes to microglia, chief of which is increased microglial activation (Fig. 2).

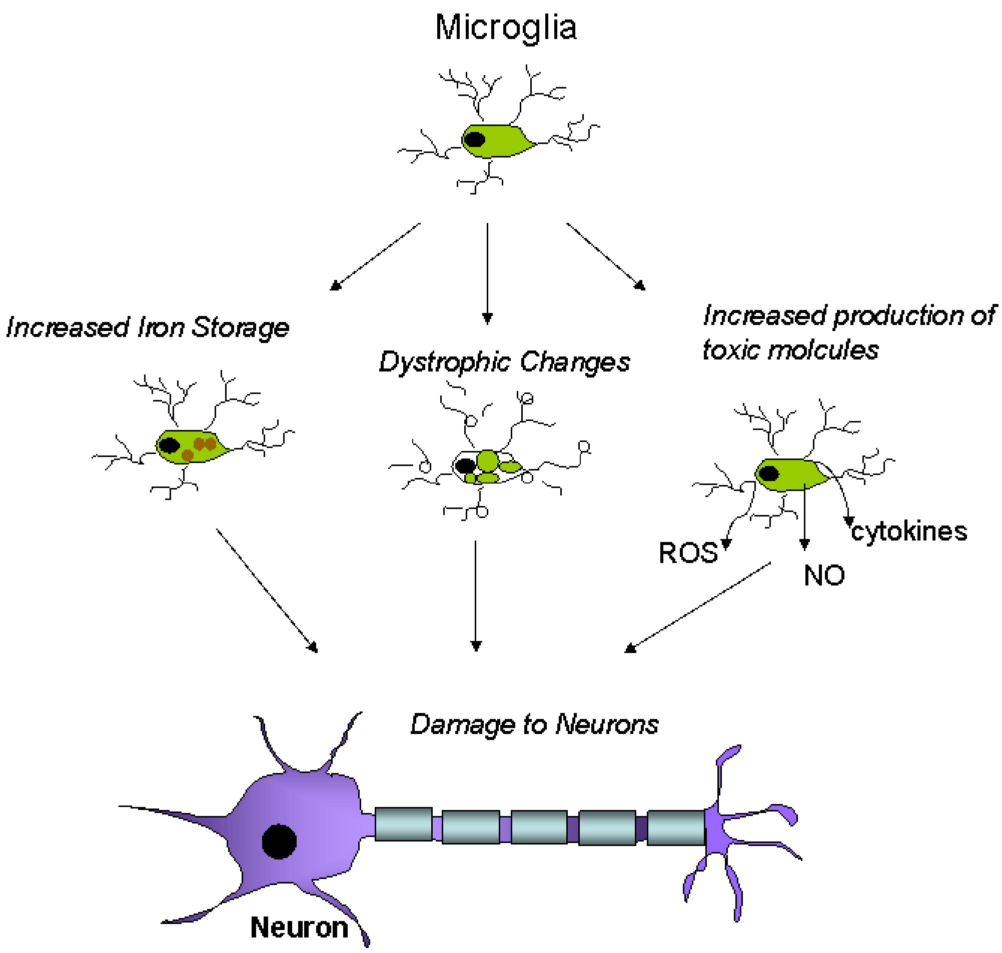

**FIGURE 2.** Age-related changes in microglia that could influence neuronal survival. Microglia in the aging brain include a high percentage of microglia that show no changes, but a proportion of microglia show a number of changes that include increased iron storage, dystrophic changes, and increased production of potentially toxic substances, such as cytokines, NO, and ROS. Microglia with increased iron storage could potentially generate increased levels of ROS through Fenton chemistry. The combination of these changes could damage neurons and induce changes associated with aging in the brain.

## CONCLUSION

Microglia are key players in neuronal health in the nervous system. It is therefore logical to suggest that the longevity of neurons requires the cooperation of microglia. If that cooperation breaks down, then it may play a significant role in both cognitive decline and other age-related changes in the central nervous system. Neurodegenerative diseases are associated with aging and their pathological process shows great similarity to the more protracted changes in aging. Increased iron storage by microglia may be sufficient to push them into a phenotype that is hostile to neurons in terms of their health and survival. This intriguing possibility opens the path to possible new models of aging in the brain. Microglia can be manipulated in culture and provide an invaluable resource for future research into the mechanisms behind all forms of neuronal damage. While studies of neurodegeneration involving microglia have flourished, the study of aging and microglia is a relative unexplored territory.
